# Traditional Chinese Medicine Enema Therapy in a Patient With a Confirmed Negative SARS-CoV-2 Test in the Respiratory Tract but Positive in the Intestinal Tract: A Case Report

**DOI:** 10.3389/fpubh.2021.687283

**Published:** 2021-07-09

**Authors:** Yuzhu Dai, Zhiyou Zhao, Huajun Zhou, Dedong Huang, Jianjun Luo, Cunhai Zhang, Qingyong Chen, Xingcan Chen, Yuan Yao, Xiaoxiao Jiang, Jun Cheng

**Affiliations:** ^1^Department of Clinical Laboratory, West Lake Hospital Affiliated to Hangzhou Medical College, Hangzhou, China; ^2^Department of Traditional Chinese Medicine, West Lake Hospital Affiliated to Hangzhou Medical College, Hangzhou, China; ^3^Department of Infection, West Lake Hospital Affiliated to Hangzhou Medical College, Hangzhou, China; ^4^Department of Critical Care Medicine, West Lake Hospital Affiliated to Hangzhou Medical College, Hangzhou, China; ^5^Department of Respiratory and Digestive Medicine, West Lake Hospital Affiliated to Hangzhou Medical College, Hangzhou, China; ^6^Department of Radiology, West Lake Hospital Affiliated to Hangzhou Medical College, Hangzhou, China; ^7^West Lake Hospital Affiliated to Hangzhou Medical College, Hangzhou, China

**Keywords:** SARS-CoV-2 infection, traditional Chinese medicine enema therapy, discharge standard, nucleic acid test of SARS-CoV-2, fecal-oral transmission

## Abstract

We report the case of a 43-year-old man who was infected with SARS-CoV-2 in February 2020 and actively cooperated with treatment in the hospital. During the course of treatment, we found that the respiratory SARS-CoV-2 nucleic acid became negative, but remained positive in the intestinal tract. As a result, we adjusted the treatment plan to include traditional Chinese medicine enema treatment. The patient had negative intestinal SARS-CoV-2 nucleic acid test within 4 days, and the subsequent repeated review of intestinal SARS-CoV-2 nucleic acid was negative, and the virus was undetectable. It is suggested that traditional Chinese medicine enema treatment may be helpful to remove the SARS-CoV-2 in the intestines of patients with COVID-19 infection, and may support the treatment of patients with respiratory SARS-CoV-2 nucleic acid negative and positive in the intestinal tract.

## Introduction

In December 2019, an unknown viral pneumonia was first reported in Wuhan, China, and subsequently reported worldwide (1–4). The virus was later identified as a novel coronavirus (SARS-CoV-2) belonging to the genus Betacoronavirus, which is ~80% similar to the severe acute respiratory syndrome coronavirus (SARS-CoV) reported in 2003 (4, 5). In response to the emerging threat posed by this virus, the World Health Organization (WHO) announced a public health emergency of international concern on January 30, 2020, and further declared a pandemic on March 11, 2020. The new virus was named SARS-CoV-2 by the Coronavirus Study Group of the International Committee for the Taxonomy of Viruses, while the resulting disease was termed coronavirus disease (COVID-19) by the WHO. As of February 24, 2021, the available WHO data show that almost all countries have reported continuous cases of SARS-CoV-2 infection.

SARS-CoV-2 is a positive-sense, single-stranded RNA virus with strong genetic similarity to bat-borne coronavirus (88%), but the intermediate host has not yet been identified. SARS-CoV-2 is the seventh coronavirus that is known to infect humans (1); among which, 229E, NL63, OC43, and HKU1 only cause symptoms of the common cold and upper respiratory tract infections (6, 7). In contrast, SARS coronavirus (SARS-CoV) in 2003, Middle East Respiratory Syndrome coronavirus (MERS-CoV) in 2012, and novel coronavirus (SARS-CoV-2) in 2019 can all be cross-infected by human-to-human transmission, thus causing large-scale atypical pneumonia (8), which leads to severe lower respiratory tract infection with acute respiratory distress syndrome (ARDS) and extra-pulmonary manifestations (9–11). The responding CD4+ cells access the small intestine through the intestinal lung shaft after the infection of lung cells by SARS-CoV-2 binding to the angiotensin converting enzyme 2 (ACE2) receptor, which leads to intestinal immune damage and diarrhea. In addition, since SARS-CoV-2 can combine with ACE2 in the intestinal mucosa, gastrointestinal symptoms may occur as a result of destruction of the intestinal mucosal barrier and release of inflammatory cytokines. Gastrointestinal symptoms are the most common complications and can cause liver damage; indeed, if not treated, they may also cause coma and circulatory failure (11, 12). Therefore, treatment regimens should be adjusted in a timely manner when patients with COVID-19 develop gastrointestinal symptoms.

The guidelines for COVID-19 issued by the National Health Council (13), and the army's support for the Hubei medical team COVID-19 diagnosis and treatment plan (14) did not require the results of SARS-CoV-2 nucleic acid in the stool as a discharge standard. However, in the consensus reached by experts on the comprehensive treatment of COVID-19 issued by Shanghai (15), it is clear that patients with COVID-19 can only be discharged after their fecal nucleic acid becomes negative. In particular, it is unclear whether negative SARS-CoV-2 nucleic acid in the respiratory tract and positive SARS-CoV-2 nucleic acid in the stool meets the discharge criteria. Here we report a patient with COVID-19 who presented with SARS-CoV-2 negative nucleic acid in the respiratory tract twice (meeting the discharge standard) and persistent positive stool after receiving emergency treatment. In this case, we used traditional Chinese medicine (TCM) enema for further treatment of this patient, which may be providing a novel way to accelerate intestinal virus cleaning. In addition, we describe the clinical characteristics of the patient before and after treatment. The details are provided below.

## Case Description

### Case Report

**Figure 1:**
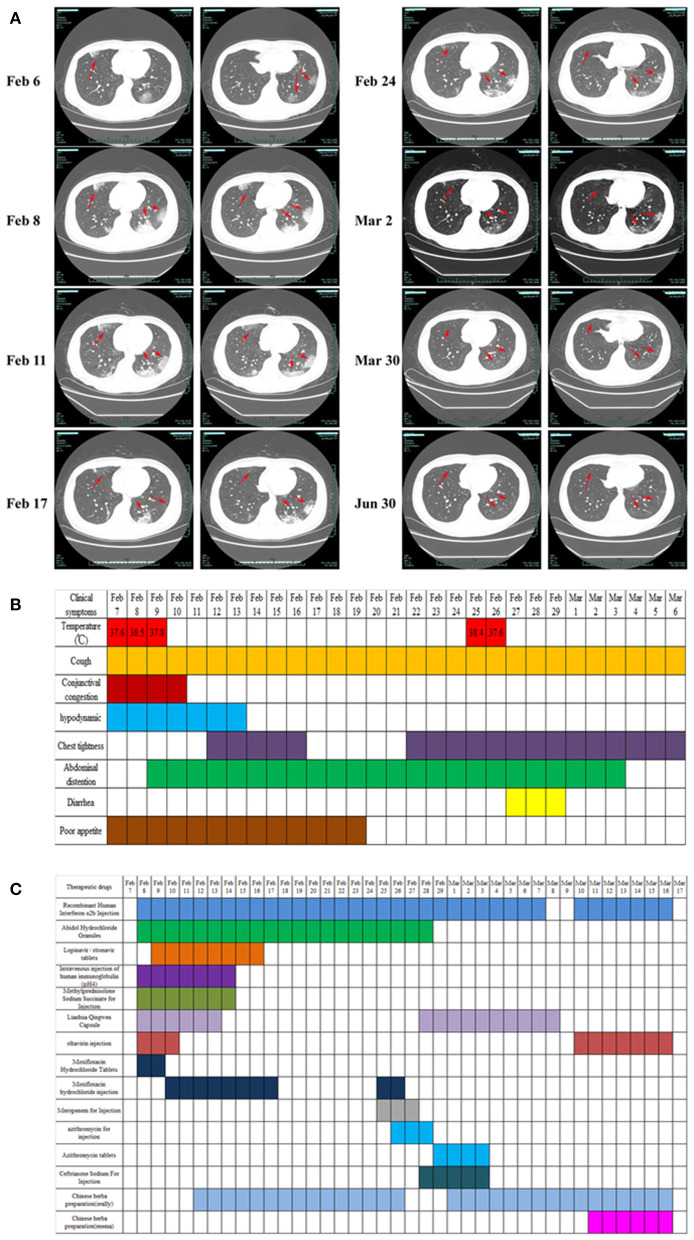
Clinical imaging, symptoms, and drug treatment data of patients with COVID-19. **(A)** CT image changes of patients with COVID-19 from first visit to discharge. **(B)** Changes in clinical symptoms in patients with COVID-19 during treatment after admission. **(C)** Medication information for patients with COVID-19 during hospitalization. Drug dosage: Recombinant human interferon α2b injection (6 million U, twice daily, aerosol inhalation); abidol hydrochloride granule (0.2 g, 3 times daily, oral administration); lopinavir/ritonavir (2 capsules, twice daily, oral administration); intravenous injection of human immunoglobulin (pH 4) (20 g, once daily, intravenous injection); methylprednisolone sodium succinate for injection (40 mg, once daily, subcutaneous injection); Lianhua Qingwen capsule (1.4 g, 3 times daily, oral administration); ribavirin injection (0.5 g, twice daily, intravenous injection); moxifloxacin hydrochloride tablets (400 mg, once daily, oral administration); moxifloxacin hydrochloride injection (0.4 g, once daily, intravenous injection); meropenem for injection (1 g, once per 8 h, intravenous injection); azithromycin for injection (0.5 g, once daily, intravenous injection); azithromycin tablets (0.5 g, once daily, oral administration); ceftriaxone sodium for injection (2 g, once daily, intravenous injection).

On February 3, 2020, a 43-year-old man developed a fever without a clear cause, and his body temperature was 38°C with no other obvious symptoms. After taking Lianhua Qingwen Capsule himself, his body temperature returned to normal. On February 5, 2020, the patient experienced fever again, with a normal body temperature after measurement, a slight cough, a small amount of white sputum, and reported being more sleepy than usual. On February 6, the patient visited the Fever Clinic of West Lake Hospital, affiliated with Hangzhou Medical College. The patient's past medical history involved generally good health, with no history of high blood pressure, diabetes or heart disease, and no travel to Wuhan, Wenzhou, or other key COVID-19 outbreak areas or exposure history. However, the patient described that he had gone to Lanxi County, Zhejiang Province, where COVID-19 had been confirmed and reported before January 22, and his sister, who lived with him at home, had cough, low fever, diarrhea, and other clinical symptoms before February 3 (later confirmed as COVID-19 by Xixi Hospital in Hangzhou). According to the above description, the patient was immediately requested to undergo chest computed tomography (CT). The CT scans showed multiple lamellar ground glass, nodular, slightly high-density shadows in both lungs, and prominent sculptural shadows. Viral pneumonia was also considered (Figure 1A). As a result, the patient was admitted to the hospital on February 7 with suspected COVID-19 (16).

Physical examination on admission showed (Figure 1B) the following: body temperature, 37.6°C; blood pressure, 129/77 mmHg; respiratory rate, 18 times/min; pulse, 82 times/min; and blood oxygen saturation, 98%. After admission, the patient's vital signs were stable, with a few coughs, a small amount of white sputum, mild congestion of the eyelid conjunctiva, pharynx redness, and no obvious tonsil enlargement. Blood tests showed a decrease in lymphocyte percentage (16.8%, normal: 20–50%) and a lymphocyte count of 1.26 × 10^9^/L (normal: 0.8–4 × 10^9^/L). The proportion of neutrophils was increased (76.6%, normal: 40–75%), the absolute value of neutrophils was normal (5.74, normal: 1.40–7.13 × 10^9^/L), and the hypersensitive C-reactive protein (CRP) was increased (10.77 mg/L, normal < 8 mg/L). The test results for influenza A and B, parainfluenza, respiratory syncytial virus, rhinovirus, adenovirus, and mycoplasma pneumoniae antigen were negative. The test results of two specimens (nasopharyngeal swabs and sputum) of SARS-CoV-2 were positive, and the preliminary diagnosis was COVID-19 (common type).

On February 7, the patient was treated with moxifloxacin hydrochloride tablets (400 mg, once daily, oral administration) to prevent infection. Abidol hydrochloride granule (0.2 g, three times daily, oral administration), ribavirin injection (0.5 g, twice daily, intravenous injection), recombinant human interferon α2b injection (6 million U, twice daily, aerosol inhalation), Lianhua Qingwen capsule (1.4 g, three times daily, oral administration) was used to clear away heat and detoxify, and human immunoglobulin (PH4) was injected intravenously (20 g, once daily) to enhance immunity. In addition, patients were treated with antipyretic and fluid supplements according to their symptoms (Figure 1C).

On February 7–9, the patient's vital physical signs were stable, with intermittent fever and blood oxygen saturation fluctuating between 93 and 99%. The patient complained of cough with white mucous sputum, poor gastric uptake, fatigue, loss of appetite, abdominal distension, and occasional retching. Re-examination chest CT showed that the lesion of the bilateral lung infection was larger and thicker than before, and the image showed significant progress compared to before (Figure 1A). On the same day, the ongoing drugs were switched to moxifloxacin hydrochloride injection (0.4 g, once daily, intravenous injection) for anti-infection, the use of lopinavir/ritonavir was increased (2 capsules, twice daily, oral administration), and methylprednisolone sodium succinate was injected (40 mg, once daily, subcutaneous injection) to reduce pulmonary interstitial edema and control the progression of the disease. The patient's third chest CT re-examination on February 11 showed no enlargement of the lesion, and the progress of the disease was basically under control (Figure 1A). Oral Chinese medicine decoction treatment was started on February 12 (the Chinese medicine prescription is shown in Table 1), and Chinese medicine diagnosed “loemia” (damp toxin epidemic). The patient's fourth chest CT on February 14 showed scattered small nodules in the right lung and some absorption of the left lung lesions (Figure 1A). The patient's respiratory symptoms improved, and his body temperature remained normal. The nucleic acid test of SARS-CoV-2 on nasopharyngeal swabs was negative, the sputum specimen was still positive, and as such, the antiviral treatment regimen was maintained.

**Table 1:** Traditional Chinese medicine prescriptions from February 12 to March 13, 2020.

**Date**	**Herb prescription**
February 12	*Radix Bupleuri* 10 g, *Bamboo Sap Pinellia* 10 g, *Radix Scutellariae* 10 g, *Radix Codonopsitis* 10 g, *Pericarpium Citri Reticulatae* 10 g, *Rehmannia Dride Rhizome* 10 g, *Radix Aucklandiae* 10 g, *Amomum villosum* 6 g, *stir-baked Fructus Setariae Germinatus* 15 g, *stir-baked Fructus Hordei Germinatus* 15 g*, prepared Radix Glycyrrhizae* 6 g; take 7 doses, once daily, oral administration.
February 19	*Broil Dwarf Yellow Daylily* 15 g, *Radix Codonopsitis* 15 g, *Radix Glehniae* 15 g, *Ophiopogonis Tuber* 15 g, *prepared Rhizome of Rehmannia* 15 g, *prepared Radix Scutellariae* 10 g, *Armeniacae Semen* 10 g, *Pericarpium Trichosanthis* 15 g, *Lignum Santali Album* 5 g, *Salvia Miltiorrhiza* 15 g, *Chinese Magnoliavine Fruit* 6 g, *Radix Scutellariae* 10 g, *Heartleaf Houttuynia Herb* 15 g, *Fasciculus Vascularis Luffae* 15 g, *Radix Glycyrrhizae* 10 g; take 7 doses, once daily, oral administration.
February 27	*Honeysuckle* 15 g, *Forsythia* 10 g, *LophatherumGracile* 10 g, *Achene of Creat Burdock* 10 g, *Schizonepeta* 10 g, *RhizomaPhragmitis* 10 g, *CoptisChinensis* 10 g, *Radix PaeoniaeRubrathe Root of Common Peony* 10 g, *Coix Seed* 60 g, *PolyporusUmbellatus* 15 g, *Rhizoma Atractylodis Macrocephalae* 20 g, *seed of Asiatic Plantain* 15 g, *prepared rhubarb* 10 g, *Radix Glycyrrhizae* 10 g; take 7 doses, once daily, oral administration.
March 6	*Radix Codonopsitis* 15 g, *Radix Pseudostellariae* 15 g, *Astragalus Membranaceus* 20 g, *Radix Aucklandiae* 10 g, *Amomum Villosum* 6 g, *Poria Cocos* 20 g, *Coptis Chinensis* 10 g, *parched white Atractylodes Rhizome* 15 g, *Pinellia Ternata* 10 g, *Pericarpium Citri Reticulatae* 10 g, *Semen Ziziphi Spinosae* 30 g, *stir-fried green beans* 15 g, *honey-fried Radix Glycyrrhizae* 6 g, *stir-fried Coix Seed* 30 g; take 7 doses, once daily, oral administration
March 10	*Rhubarb* 10 g, *Mangnolia Officinalis* 20 g, *Radix Scutellariae* 15 g, *CoptisChinensis* 10 g, *Radix Glycyrrhizae* 10 g, *fruit of Citron or Trifoliate Orange* 20 g; take 3 doses, once daily, enema therapy.
March 13	*Radix Codonopsitis* 20 g, *Astragalus Membranaceus* 30 g, *Radix Pseudostellariae* 10 g, *Amomum Villosum* 6 g, *Bighead Atractylodes Rhizome* 15 g, *Poria Cocos* 15 g, *stir-fried green beans* 15 g, *Coix Seed* 30 g, *Salvia Miltiorrhiza* 30 g, *Agastache rugosu* 10 g, *Eupatorium* 10 g, *Pinellia Ternata* 10 g, *Pericarpium Citri Reticulatae* 10 g, *prepared Radix Glycyrrhizae* 10 g; take 7 doses, once daily, oral administration.
March 13	*Rhubarb* 15 g, *Mangnolia Officinalis* 20 g, *Fruit of Citron or Trifoliate Orange* 15 g, *Coptis Chinensis* 15 g, *Radix Scutellariae* 15 g, *Radix Glycyrrhizae* 6 g; take 3 doses, once daily, enema therapy.

On February 17 and February 24, the fifth and sixth chest CT scans showed that the scope of the lung lesions narrowed and gradually absorbed. The white blood cell (WBC) count, lymphocyte count, and high-sensitivity CRP (hs-CRP) levels were normal. In the evening of February 25, the patient had a fever with a body temperature of 38.2°C. The sputum and feces of SARS-CoV-2 nucleic acids were all positive. The blood routine examination showed that the WBCs were elevated (9.82 × 10^9^/L, normal, 4.0–10.0 × 10^9^/L), the neutrophils were increased (9.05 × 10^9^/L, normal: 1.40–7.13 × 10^9^/L), the lymphocyte count was decreased (0.50 × 10^9^/L, normal: 0.8–4 × 10^9^/L), and the erythrocyte sedimentation rate was increased by 55 mm/h (normal: 0–15 mm /h). According to the results of laboratory examination, considering bacterial infection, meropenem (1 g, once per 8 h, intravenous injection), azithromycin for injection (0.5 g, once daily, intravenous injection) combined with anti-infection, and abidol hydrochloride granules (0.2 g, three times daily, oral administration), recombinant human interferonα-2B injection (6 million U, twice daily, aerosol inhalation) were added to continue the antiviral treatment. On February 27, the patient's temperature returned to normal. On February 29, SARS-CoV-2 nucleic acid (nasopharyngeal swab, sputum, urine, blood) was negative, but diarrhea symptoms appeared (from February 27–29, stopping TCM decoction), and chest tightness was more obvious than before. Azithromycin was discontinued considering the side effects. Azithromycin tablets (0.5 g, once daily, oral administration) and ceftriaxone sodium for injection (2 g, once daily, intravenous injection) were used for anti-infection, and Huangqi Shengmai drink (10 mL, three times daily, oral administration) was added for the treatment of chest tightness. On March 3, diarrhea and chest tightness were better than before. The patient's seventh chest CT re-examination showed that the focus of pulmonary infection was further absorbed than on February 24, and the scope of the focus continued to narrow. Symptomatic treatment and TCM adjuvant treatment were continued. On March 9, the sputum SARS-CoV-2 nucleic acid was recovered, the stool nucleic acid was positive, and other specimens (urine, blood, and throat swabs) were negative. The antiviral regimens were continued, and Chinese medicine enema was used to promote the elimination of SARS-CoV-2 and promote the negative conversion of SARS-CoV-2 nucleic acid in the gut. The detailed medication plans are shown in Table 1 and Figure 1C. On March 17, three consecutive re-examinations of the respiratory tract and feces showed SARS-CoV-2 nucleic acid as being negative. The patient's body temperature remained normal for more than 3 days, and re-examination of chest CT showed improvement in absorption. According to the latest version of the Diagnosis and Treatment Protocol for Novel Coronavirus Pneumonia of China, patients who meet the following criteria can be discharged (http://www.nhc.gov.cn/xcs/fkdt/202002/54e1ad5c2aac45c19eb541799bf637e9.shtml), and the patient was discharged into the isolation point for follow-up observation.

### Imaging Examination

From the first chest CT examination on February 6, 2020, to the re-examination after discharge, the patient underwent a total of eight chest CT scans. In the early stage of the disease (February 6–8), CT showed that the lung lesions had imaging manifestations of continuous progress. From February 11, CT showed that the lesions gradually shrunk. On March 30, the re-examination CT showed that the lung images had returned to normal (Figure 1A).

### Laboratory Examination

**Figure 2:**
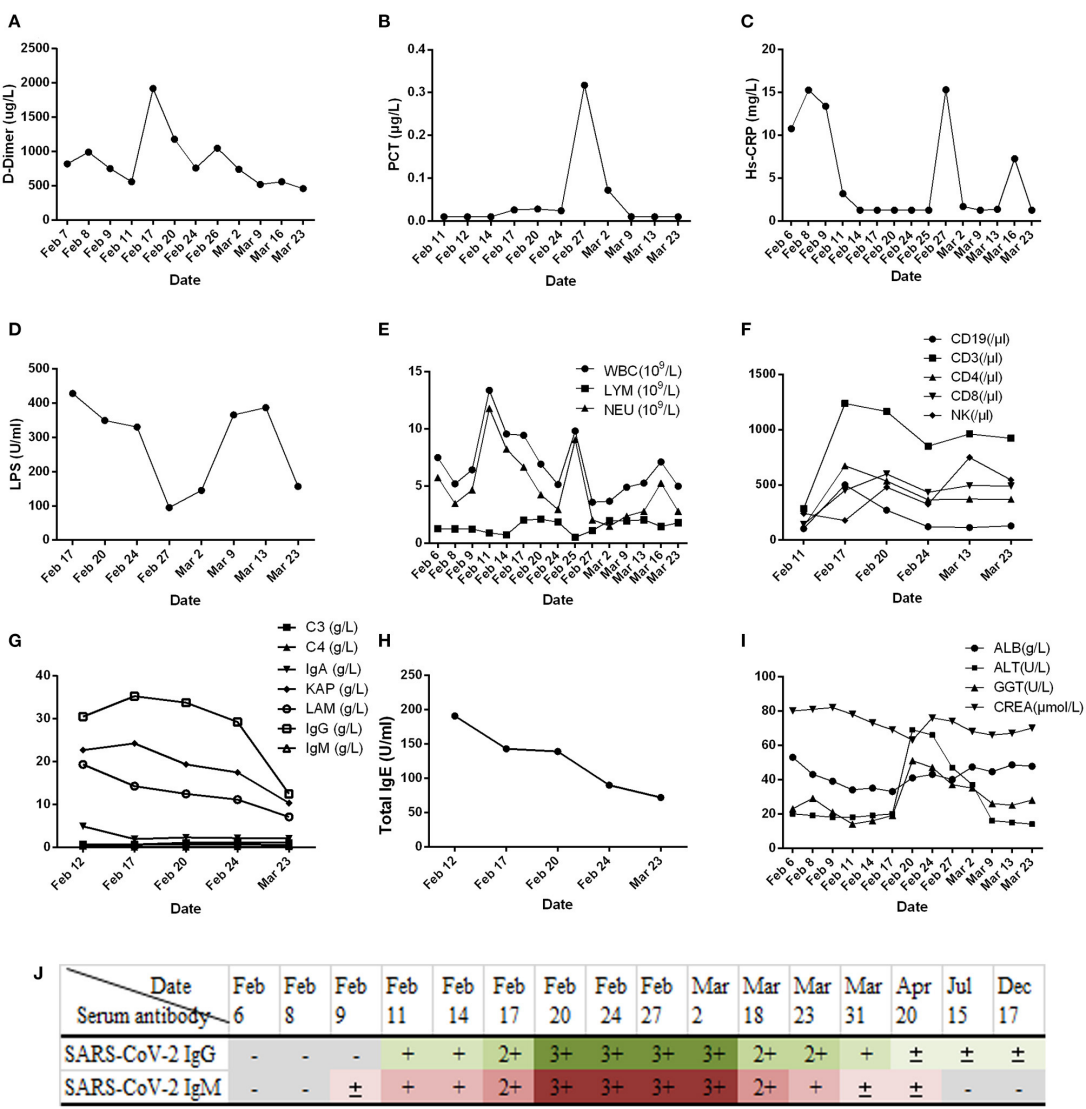
Results of routine laboratory examination of patients with COVID-19 during admission. **(A)** Plasma D-dimer; **(B)** serum procalcitonin (PCT); **(C)** high-sensitivity C-reactive protein (Hs-CRP); **(D)** lipopolysaccharide (LPS); **(E)** white blood cell count; **(F)** classification count of lymphocyte subsets; **(G)** complement (C3, C4), immunoglobulin (IgA, IgG, IgM), and light chain (KAP, LAM: Immunoglobulin light chain kappa and lambda); **(H)** immunoglobulin E (IgE); **(I)** liver (ALB, albumin; ALT, alanine aminotransferase; GGT, gamma glutamyl transpeptidase) and kidney function (CREA, creatinine); **(J)** results of serum SARS-CoV-2 IgG/IgM detection in patients with COVID-19. The SARS-CoV-2 serum antibody (IgG/IgM) test kit (colloidal gold method) was provided by Zhuhai Lizhu Reagent Co., Ltd.; “–” represents negative, “±” represents weak positive, and “+, 2+, 3+” represents positive, and it was enhanced step by step.

**Figure 3:**
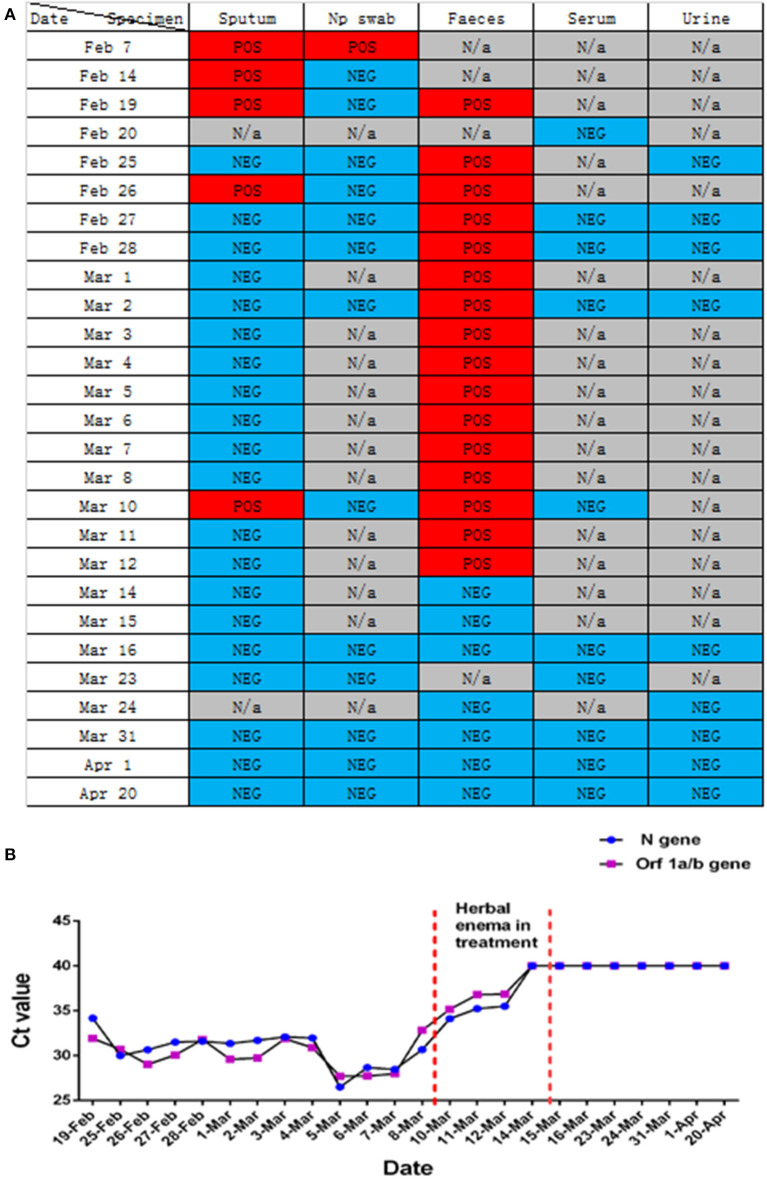
Changes in nucleic acid detection in various patients with COVID-19. **(A)** Detection of SARS-CoV-2 nucleic acids in different samples of patients with COVID-19 during hospitalization. POS, Test result was positive; NEG, Test result was negative; N/a, No relevant tests were carried out. **(B)** Detection of SARS-CoV-2 nucleic acid in the feces of patients with COVID-19 pneumonia during hospitalization. Before February 25, 2020, the New Coronavirus 2019-nCOV nucleic acid detection kit (real-time fluorescent PCR method) provided by Shanghai Berger Biotechnology Co., Ltd. was used, and the New Coronavirus 2019-nCOV nucleic acid detection kit (real-time fluorescence PCR method) provided by Da'an gene Limited by Share Ltd. of Zhongshan University was used after February 25, 2020.

Clinical samples, nasopharyngeal swabs, feces, urine, whole blood, serum, and sputum were collected. Routine tests were carried out on the day of admission, including blood; sputum; throat swab culture (fungi and bacteria); blood routine (cell count and proportion); liver function [albumin (ALB), alanine aminotransferase (ALT), gamma glutamyl transpeptidase (GGT)]; renal function; inflammatory indexes [hs-CRP, lipopolysaccharide (LPS), procalcitonin (PCT)]; and coagulation function (D-dimer). In addition, common acute respiratory pathogens (influenza A virus, influenza B virus, adenovirus, parainfluenza, and syncytial virus), coronavirus (MERS, SARS, 229E, NL63, OC43, and HKU1), and SARS-CoV-2 nucleic acids were detected. Following identification of the pathogen of infection, serum SARS-CoV-2 antibody, cellular immunity, and humoral immunity were detected. Disease progression was observed by continuous sample collection (Figures 2, 3).

## Discussion

The outbreak of SARS-CoV-2 from December 2019 to the present has been catastrophic, has seriously damaged the global public health system, and continues to pose a serious threat to the health of humans worldwide (17–19). With the deepening of the research on the clinical characteristics and mechanism of SARS-CoV-2 infection, many studies have confirmed that SARS-CoV-2 and SARS-CoV have similar infection mechanisms (20, 21), mainly through the ACE2 receptor to infect target cells. Moreover, the main symptoms after infection are respiratory system reactions, while gastrointestinal symptoms are also very common. Patients with COVID-19 accompanied by gastrointestinal symptoms are more likely to experience ARDS and liver damage with an inadequate prognosis (10, 22). Although there is no direct evidence to prove the fecal oral transmission of SARS-CoV-2, existing studies have shown that SARS-CoV-2 can be detected in the respiratory tract and fecal samples of most patients with COVID-19 RNA (22–24). In addition, it has been proven that SARS-CoV-2 can be isolated from the feces of patients with COVID-19 (25, 26), which further reflects the risk of fecal oral transmission of SARS-CoV-2 (27), and may pose new challenges to the control and prevention of COVID-19. Here we report a case of COVID-19 with negative conversion of SARS-CoV-2 nucleic acid in the respiratory tract and persistent positivity in the intestinal tract. In order to speed up the clearance of SARS-CoV-2 in the intestinal tract and reduce the risk of fecal oral transmission, we used a TCM enema for treatment and achieved good results.

In this study, we report that patients with COVID-19 developed rapidly in the early stage of the disease (Figure 1A). We added methylprednisolone sodium succinate (40 mg, once daily, subcutaneous injection) in the clinic, which effectively controlled the disease progression, indicating the effectiveness of appropriate glucocorticoid treatment in the early stage (28). In addition, the laboratory results (Figures 2F–H) also indicate that the humoral and cellular immunity of patients is active, and each index is at a high level in the early stage of infection. This may be related to immune disorders caused by the early progression of the disease and induction of cellular immune factors, which is consistent with previous reports (29, 30). With improvement of the disease, each index of the immune system shows a decline. In particular, the high level of IgE (Figure 2H) indicates that there is a certain degree of type I allergy in COVID-19 (31). Indeed, anti-SARS-CoV-2 IgM antibody can be detected as early as 1 week after the onset of the disease, and anti-SARS-CoV-2 IgG antibody is produced relatively rapidly. Anti-SARS-CoV-2 IgM and anti-SARS-CoV-2 IgG reached their peak 3 weeks after infection, and, as of December 2020, the anti-SARS-CoV-2 IgG antibody in patients was weakly positive (Figure 2J), which was consistent with that reported in the literature (24). During treatment, the patient developed fever again, and the laboratory results (Figures 2A–E) showed an increase in inflammatory indices (Hs-CRP, D-dimer, WBC count, PCT), which indicated that the patient had a secondary pulmonary infection. At the same time, the secondary infection induced positive conversion of SARS-CoV-2 RNA in sputum samples (Figure 3A; February 26). In view of this phenomenon, combined antiviral and anti-infection treatments should be considered (Figure 1C). Therefore, in the process of treatment and nursing, secondary infection of the lung must be prevented and controlled. In the current case, the patient's sputum samples were negative for SARS-CoV-2 nucleic acid on February 27 and 28 (Figure 3A; February 27 and 28). At this time, antiviral drugs were stopped, and only inhaled interferon was maintained. On March 9, the patient again tested positive for SARS-CoV-2 nucleic acid in the sputum and remained positive for SARS-CoV-2 nucleic acid in the stool (Figure 3A, from February 19 to March 9). According to the analysis of previous literature, the apparent re-emergence of SARS-CoV-2 nucleic acid in the sputum may be related to the persistent positive SARS-CoV-2 in the feces (32). Therefore, we adjusted the treatment plan again, and TCM enema treatment was added. After treatment with the TCM enema, the threshold cycle value (Ct-value) of the virus gradually increased to > 40 cycles (Figure 3B), and patients with COVID-19 who met the discharge criteria were discharged from the hospital (33). After repeated re-examination, SARS-CoV-2 RNA in feces, sputum, urine, and blood samples was negative.

With the outbreak of SARS-CoV-2, medical experts worldwide have a new understanding of the role of TCM in the treatment of infectious diseases. Multiple studies have reported the efficacy of TCM enema in other diseases, such as epidemic encephalitis B (34), hand foot mouth disease (35), and viral hepatitis (36, 37). We focused on the clinical manifestations of this patient: aversion to cold, fever, sore throat, white phlegm, chest tightness, dry stool, white, and greasy tongue coating. Based on the dialectical system of viscera in TCM, the lung and large intestine interact with each other, and the residual toxin of lung heat moves down to the large intestine. The syndrome types in the patients were dampness heat stagnation of the lung and heat stagnation of the large intestine. TCM *Coptis Chinensis* and *Radix Scutellariae* can clear heat and dampness, relieve fire, and detoxify; *rhubarb* can break accumulation and remove heat between intestines; and *fruit of citrus or trifoliate orange* and *Magnoliae Officinalis* can disperse dampness and remove distension. The corresponding enema scheme was formulated to allow the medicine to directly reach the disease center (Table 1). After 4 days of TCM enema treatment, the intestinal stool SARS-CoV-2 was negative. Many studies have reported variations in the time of SARS-CoV-2 turning negative in the feces of patients with COVID-19 (38, 39), which is related to different treatment schemes and different immune responses to the virus. However, it is clear that the nucleic acid in the feces of most patients with COVID-19 lasts longer than that in the respiratory tract. According to the laboratory nucleic acid test results (Figures 3A,B). On March 10, the nucleic acid of sputum samples returned to positive, indicating that the infection process of the patient was still repeated, and then the patient received TCM enema treatment. The viral intestinal nucleic acid showed an obvious downward trend (Ct value suddenly increased) during the treatment. It indirectly reflects that SARS-CoV-2 may be effectively removed from the intestine with the use of drugs, was effectively removed from the intestine, which may effectively shortened the intestinal clearance time of the virus. However, this study only used TCM enema for one patient with COVID-19, and did not set up a control case. Therefore, regardless of TCM enema is effective for different patients with COVID-19, and the mechanism of TCM enema accelerating intestinal virus clearance need to be further studied.

In summary, although some vaccines against SARS-CoV-2 have been used clinically, including inactivated SARS-CoV-2, mRNA, and recombinant adenovirus vaccines (40, 41), due to the long development cycle of vaccines and other new drugs, it is still an effective method to cut off the source of infection. TCM enema therapy has been shown may accelerate the clearance of SARS-CoV-2 in the intestines of patients with COVID-19, shorten the positive time, and realize the negative effect of feces during hospitalization. The negative fecal nucleic acid can be further used as the discharge standard for patients with COVID-19 after hospitalization to prevent fecal oral transmission.

## Data Availability Statement

The original contributions presented in the study are included in the article/supplementary material, further inquiries can be directed to the corresponding authors.

## Ethics Statement

Written informed consent was obtained from the individual(s) for the publication of any potentially identifiable images or data included in this article.

## Author Contributions

YD and JC conceived the study, participated in its design, drafted and revised the manuscript for content including medical writing for content, analysis, and interpretation of data. ZZ, HZ, DH, JL, CZ, QC, XC, and YY conceived the study, participated in its design managed microbiology laboratory assays, revised the manuscript, and collected TCM treatment prescription. XJ was responsible for the collection and collation of clinical data. All authors read and approved the final manuscript.

## Conflict of Interest

The authors declare that the research was conducted in the absence of any commercial or financial relationships that could be construed as a potential conflict of interest.
